# A Routine Coronary Angiography before Carotid Endarterectomy as an Example of Interdisciplinary Work: The Immediate Results of the Surgery

**DOI:** 10.3390/jcm13185495

**Published:** 2024-09-17

**Authors:** Alexey N. Sumin, Anna V. Shcheglova, Olesya V. Adyakova, Darina N. Fedorova, Denis D. Yakovlev, Natalia A. Svinolupova, Svetlana V. Kabanova, Anastasia V. Malysheva, Marina Yu Karachenko, Vasily V. Kashtalap, Olga L. Barbarash

**Affiliations:** 1 Federal State Budgetary Scientific Institution “Research Institute of Complex Problems of Cardiovascular Diseases”, Kemerovo 650002, Russia; nura.karpovitch@yandex.ru (A.V.S.); adyjakova.lesya@mail.ru (O.V.A.); fedorova.darina.2001@mail.ru (D.N.F.); v_kash@mail.ru (V.V.K.); olb61@mail.ru (O.L.B.); 2 Federal State Budgetary Educational Institution of Higher Education “Kemerovo State Medical University” of the Ministry of Health of the Russian Federation, Kemerovo 650056, Russia

**Keywords:** carotid endarterectomy, coronary angiography, perioperative complications, risk assessment, obstructive lesion of the coronary arteries, myocardial revascularization

## Abstract

**The aim**: to evaluate the incidence of obstructive lesions of the coronary arteries during routine coronary angiography (CAG) before carotid endarterectomy (CEA) and the incidence of perioperative complications. **Materials and Methods**: We examined a continuous sample of 498 patients before CEA who underwent an invasive evaluation of the coronary bed during CAG. Depending on the hemodynamic significance of coronary artery lesions, the patients were divided into three groups: group I—obstructive coronary artery disease (≥70%) (*n* = 309, 62.0%); group II—non-obstructive lesions of the coronary arteries (<70%) (*n* = 118, 23.7%); group III—intact coronary arteries (*n* = 71, 14.3%). The groups were compared with each other according to the data of the preoperative examination (clinical and anamnestic parameters, laboratory data and results of echocardiography), as well as according to the immediate results of the operation. In the hospital period, adverse cardiovascular events were assessed: death, myocardial infarction (MI), stroke, arrhythmias, atrial fibrillation or flutter (AF/AFL) and combined endpoint. **Results**: The groups differed significantly in the presence of symptoms of angina pectoris, myocardial infarction and myocardial revascularization procedures in their medical history and in the presence of chronic ischemia of the lower extremities. However, in the group of intact coronary arteries, the symptoms of angina were in 14.1% of patients, and a history of myocardial infarction was in 12.7%. Myocardial revascularization before CEA or simultaneously with it was performed in 43.0% of patients. As a result, it was possible to reduce the number of perioperative cardiac complications (mortality 0.7%, perioperative myocardial infarction 1.96%). **Conclusions**: The high incidence of obstructive lesions in the coronary arteries in our patients and the minimum number of perioperative complications favor routine CAG before CEA.

## 1. Introduction

When performing non-cardiac operations, cardiac complications are the main cause of death [1]. Therefore, the preoperative assessment of the risk of cardiac complications remains an urgent task in clinical medicine, requiring a multidisciplinary approach. On the one hand, it is necessary to identify patients with the highest risk of such complications in order to reduce the likelihood of developing fatal complications in the pre- and perioperative periods. On the other hand, it is during the preoperative examination before non-cardiac operations that the largest number of examinations are performed that do not reveal cardiac pathology [2,3]. Accordingly, it is necessary to maintain a balance between necessary and unnecessary examinations in this category of patients, which is served by periodically updated national expert recommendations [4,5,6]. In particular, the latest edition of the ESC recommendations was adopted at the congress in 2022 [7].

However, existing publications cannot resolve all controversial issues regarding the examination of patients before non-cardiac operations. One of these categories are patients who are planning operations on large and peripheral vessels, in particular, carotid endarterectomy (CEA). On the one hand, existing recommendations classify such operations as having a low or medium risk of cardiac complications (depending on the presence of cerebrovascular symptoms) [7]. On the other hand, it is recognized that the most commonly used scale for assessing the preoperative risk of cardiac complications (RCRI) underestimates the risk of cardiac complications [8], and it is proposed to use specialized scales for vascular operations [9]. In addition, a number of publications in previous years [10,11,12] indicate the advisability of routine coronary angiography (CAG) in patients before CEA surgery. However, in the diagnosis of coronary artery disease, one must take into account the fact that obstructive lesions of the coronary arteries during coronary angiography are detected only in 40% of cases according to register data [13], and the results of the ISCHEMIA study showed that even in the presence of such lesions, myocardial revascularization does not lead to improvement in patient prognosis [14]. Accordingly, modern recommendations for the diagnosis of coronary artery disease do not propose a separate scenario for examination before vascular operations, and the traditional approach of assessing pre-test, clinical probability and non-invasive tests [15] cannot be used in patients with asymptomatic coronary artery disease. Recently, many non-cardiac surgeries previously classified as high cardiac risk have been shown to have a lower risk of cardiac complications [16]. Accordingly, it has been suggested that models estimating the cardiac risk of non-cardiac surgery require periodic updating as outcomes improve [17]. To resolve these controversial issues, the present study was conducted, the purpose of which was to evaluate the frequency of detection of obstructive lesions in the coronary arteries during routine CAG before CEA surgery, as well as the frequency of perioperative complications in this cohort of patients.

## 2. Subjects, Materials, and Methods

### 2.1. Study Population

**Figure 1:**
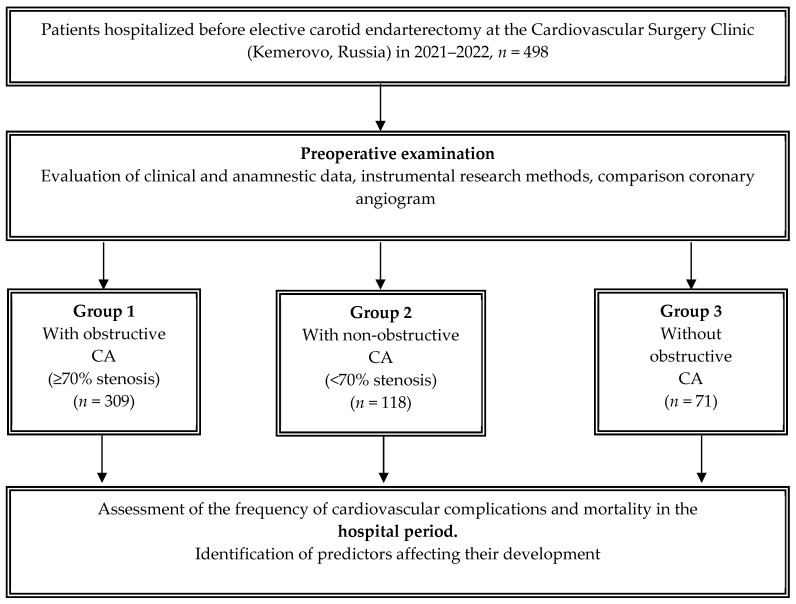
Patient flowchart. Notes: CA—coronary artery.

Consecutive patients who underwent elective CEA surgery at the cardiovascular surgery and neurosurgery departments of the Research Institute of KPSSZ for the period from 1 January 2021 to 31 December 2022 were included in a cohort, comparative, retrospective study. The study protocol was approved by the Local Ethics Committee of the Institution. All patients filled out informed consent forms to participate in the study. All patients underwent anthropometric, clinical, instrumental and laboratory examinations, the data of which were summarized and analyzed. The atherosclerotic lesion of the carotid region was verified using carotid ultrasonography (US). The significance of carotid artery stenosis was confirmed using computed tomography angiography (CTA) and/or angiographic examination. At the place of residence or in the hospital, as a non-invasive method for diagnosing coronary artery disease and in order to determine exercise tolerance, patients underwent imaging and stress tests if necessary. All patients underwent coronary angiography (CAG) using an INNOVA 3100 angiographic device (USA) before CEA. Based on the results of coronary angiography, the following groups were identified depending on the hemodynamic significance of coronary artery lesions: obstructive coronary artery lesions (≥70%); non-obstructive coronary artery lesions (<70%); and intact coronary arteries (Figure 1).

The choice of surgical treatment tactics was made by a multidisciplinary team (cardiovascular surgeon, endovascular surgeon, cardiologist, neurologist and anesthesiologist) based on clinical and instrumental data, the significance of coronary and carotid atherosclerosis, existing clinical guidelines and internal algorithms of the institution.

### 2.2. Data Collection

The information was collected retrospectively from the institutional database. Before performing CEA, laboratory (glucose, creatinine, lipid profile) and echocardiographic parameters (dimensions, volume parameters of left and right ventricle, left ventricular ejection fraction, left ventricular diastolic parameters) were assessed in all patients. The diagnosis of peripheral atherosclerosis of the arteries of the lower extremities was established in the presence of symptoms of intermittent claudication, confirmed using US data.

### 2.3. Study Outcomes (Perioperative Complications)

As significant postoperative complications of CEA, death, myocardial infarction (MI), stroke and atrial fibrillation/flutter were taken into account. We also analyzed combined endpoint (death, myocardial infarction (MI), stroke, atrial fibrillation/flutter) during the hospital stay.

### 2.4. Statistical Analyses

Statistical processing was carried out using the SPSS 17.0 software package (IBM, Armonk, NY, USA). The distribution of quantitative data was tested using the Shapiro–Wilk criterion. Considering that the distribution of all quantitative characteristics differed from normal, they are presented as the median, upper and lower quartiles. The Kruskal–Wallis test was used to compare three groups, and the Mann–Whitney test was used for the pairwise comparisons of groups. The Bonferroni correction was used to solve the problem of multiple comparisons. Qualitative values were presented in absolute numbers (n) and percentages (%), and comparisons between the groups were performed using χ^2^ tests. In the case of a small number of observations, Fisher’s exact test with Yates’ correction was used. To assess the factors associated with the combined endpoint after CEA binary logistic regression analysis (the forward stepwise LR method) was used. Differences were considered significant at (*p*) less than 0.05.

## 3. Results

A total of 498 patients aged 46 to 86 years with significant carotid artery stenosis were consecutively included in this study. Based on the results of coronary angiography, depending on the hemodynamic significance of coronary artery lesions, the patients were divided into three groups: group I—obstructive coronary artery lesions (≥70%) (*n* = 309, 62.0%); group II—non-obstructive coronary artery lesions (<70%) (*n* = 118, 23.7%); group III—intact coronary arteries (*n* = 71, 14.3%).

The clinical and anamnestic characteristics of the compared groups are presented in Table 1. Men prevailed in the entire sample (68.7%). Arterial hypertension was recorded in 86.5% of patients, and a history of stroke was observed in 40% of cases. Patients with obstructive and non-obstructive coronary artery lesions were statistically significantly older than patients with intact coronary arteries (*p* < 0.05).

In patients with obstructive coronary artery disease, angina pectoris, a history of myocardial infarction and a history of myocardial revascularization were statistically significantly more frequently registered compared to the group of patients with non-obstructive and pure coronary arteries (*p* < 0.001). The groups had no significant differences in the prevalence of comorbid pathology (diabetes mellitus, chronic kidney disease, COPD, history of cancer). A high prevalence of lower extremity artery stenosis was noted in patients with obstructive coronary artery disease (20.3%) than in groups without obstructive coronary artery disease 11.1% (*p* = 0.026) and intact coronary arteries—8.2% (*p* = 0.0015). Noninvasive imaging tests were performed only in 9.0% of cases and the stress test was only performed in 1.0% of cases, mainly in groups with affected coronary arteries.

No significant differences were found in the intergroup analysis of biological marker concentrations at the hospital stage. Patients in all groups showed an increase in the concentration of total cholesterol, triglycerides and low-density lipoproteins, and a decrease in the content of antiatherogenic high-density lipoprotein (Table 2).

**Figure 2:**
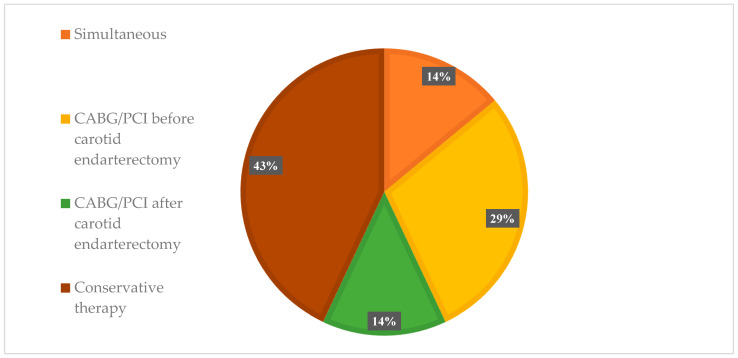
Managing patients with obstructive CAD and carotid artery disease (*n* = 309).

When analyzing the echocardiography data, it was found that the median left ventricular ejection fraction in all groups was within the normal values. But in the group with coronary artery damage ≥70%, it was statistically significantly lower (Me 62.0%) compared with the group with coronary artery damage <70% (Me 65.0%; *p* < 0.001) and clean coronary arteries (Me 63.5%; *p* = 0.015). The indicators characterizing the size of the left ventricle were statistically significantly higher in the groups with coronary artery damage compared with the group of clean coronary arteries (*p* < 0.05; Table 3). In the prehospital period, the frequency of drug therapy was low in the entire sample (Figure 2): β-blockers—62.2% of patients; angiotensin-converting enzyme inhibitors (ACE)/angiotensin receptor blockers (ARBs)—69.5% of patients; statins—72.1% of patients; aspirin—59.8% of patients.

The surgical strategy in the choice of combined and staged approaches to cerebral and myocardial revascularization is presented in Figure 2. As a result of damage to the carotid and coronary arteries, it was determined that the following tactics should be used in the group with obstructive coronary artery disease: in 42 (13.6%) patients, simultaneous intervention on the carotid and coronary pool; in 90 (29.1%) patients, myocardial revascularization was performed as the first stage before CEA; in 42 (13.6%) patients, myocardial revascularization was planned as the next stage after CEA. In the remaining cases, the conservative treatment of the coronary arteries was indicated.

The perioperative characteristics are presented in Table 4. The overwhelming majority of patients underwent classical CEA with plastic surgery of the reconstruction zone using a xenopericardial patch. The groups were comparable in terms of surgery duration and intraoperative blood loss. In the group with hemodynamically significant coronary artery lesions, 15 (4.9%) patients underwent combined CABG + CEA and 27 (8.7%) patients underwent hybrid PCI + CEA. In the group with intact coronary arteries, one case (1.4%) underwent simultaneous intervention on the carotid artery and aortic valve. An analysis of clinical scales in assessing the risk of non-cardiac surgeries had statistically significant differences only in relation to the RCRI index, with higher values in the group with coronary artery lesions ≥ 70% than in the group with coronary artery lesions <70% and clean coronary arteries (*p* < 0.001). In analyzing the incidence of adverse events that occurred during the hospital observation period (Table 5), it was found that fatal outcome occurred in two (0.4%) cases only in the group with obstructive coronary artery disease (*p* = 0.541). The cause of fatal outcome in the first case was hemorrhagic complication after simultaneous surgery (CABG + CEA); in the second case, fatal myocardial infarction developed after incomplete myocardial revascularization at the first stage in a patient with multivessel coronary artery disease. Only in the group with obstructive coronary artery disease were six (1.9%) cases of non-fatal MI recorded. Upon detailed analysis, in three cases, MI developed in patients who were prescribed conservative tactics in relation to coronary arteries. In two cases, non-fatal MI developed during combined surgery (CEA + CABG and CEA + PCI). In one case, MI developed in a patient with a certain revascularization strategy as the next stage after CEA. ACVE developed in five (1.6%) cases in the group with obstructive coronary artery disease and in one (1.4%) case in the group with pure coronary arteries (*p* > 0.05). In general, in patients with obstructive coronary artery disease, the development of a combined endpoint (fatal outcome, paroxysm of AF/AT, non-fatal MI and stroke) was statistically significantly more frequent (6.2% of cases) compared with the group with non-obstructive coronary artery disease (*p* = 0.005).

In a univariate analysis of factors associated with the development of the combined endpoint in the postoperative period, only nine parameters had *p* values less than 0.2 and they were analyzed in the multiple binary logistic regression model using the Enter method (Supplementary Table S1). The Hosmer–Lemeshow test results are shown in Supplementary Table S2; the Chi-square values were 9.716 and *p* = 0.286. These values indicate that the logistic regression is a good fit. Accordingly, these parameters were further analyzed in multiple binary logistic regression using the Forward Stepwise Likelihood Ratio method to identify independent predictors of the combined endpoint.

In a multiple binary logistic regression model (forward LR method), the following factors had a significant association (χ^2^(3) = 18.18; *p* < 0.001, Supplementary Table S3) with combined endpoint development: the presence of coronary artery disease (B = −1.214; *p* = 0.022), the presence of chronic kidney disease (B = 1.623; *p* = 0.005) and the presence of obstructive coronary artery lesions (B = −1.708; *p* = 0.014) (Table 6). This model explains only 15.3% (Nagelkerke R^2^, Supplementary Table S4) of the variance in combined endpoints and correctly classified 95.9% of cases (Supplementary Table S5). The Hosmer–Lemeshow test results at all steps of the forward LR method analysis (Supplementary Table S6) indicate that the logistic regression is a good fit in this case as well. Supplementary Table S7 presents the data from the Receiver operating characteristic curve analysis for the performance of baseline parameters in discriminating combined endpoint development after carotid endarterectomy. The area under the curve was largest for the presence of obstructive coronary artery lesions indicator (0.665, *p* = 0.012). 

## 4. Discussion

When performing CAG before CEA, obstructive coronary artery lesions were detected in 62.0% of patients. Myocardial revascularization was performed before or simultaneously with CEA in 42.7% of patients. As a result, it was possible to reduce the number of perioperative cardiac complications (mortality 0.7%, perioperative myocardial infarction—1.9%), which is below the average figures for such operations [18].

It should be noted that the percentage of patients with obstructive coronary artery disease was significantly higher than in many previously conducted studies in other centers. Thus, a review by the ESC working group indicated that obstructive coronary artery disease occurs in 25–35% of cases in patients with carotid artery stenosis [19]. The results of the study by Illuminati G et al. are consistent with these data: in the group of routine CAG before CEA, the frequency of obstructive coronary artery disease was 31.5% [20]. In this study, unlike the present one, patients without a history of coronary artery disease and without changes in echocardiography and ECG were included (i.e., the examined patients were asymptomatic in terms of cardiac pathology). A distinctive feature of our clinic is the high incidence of concomitant coronary artery disease during CEA, a trend that has persisted for a long time in our early studies. The incidence of obstructive coronary artery disease in such patients was more than 60% [21]. When assessing the fractional flow reserve during CT coronary angiography in patients before CEA in the absence of coronary artery symptoms, coronary artery lesions were detected in 57%, including those with extensive ischemia in 44% of cases [22]. In the study by Squizzato et al. [23], obstructive coronary artery disease was detected in 65.1% of patients with bilateral carotid artery stenosis >70%. Similar data were obtained when examining patients before carotid artery stenting. Coronary artery disease was detected preoperatively in 64% of cases, coronary revascularization was indicated in 41% of cases and preoperative coronary revascularization was performed in 34%. As a result, perioperative coronary ischemic complication did not develop in any cases [24]. The results of the abovementioned studies are quite consistent with our data. Apparently, the frequency of detection of coronary artery lesions depends both on the profile of the department (in our case, it is a multidisciplinary hospital which seems to lead to the concentration of patients with combined pathology) and on the preferential ways of detecting carotid stenosis (screening assessment during observation by a neurologist or the same screening in cardiac patients), as well as on the severity of carotid artery lesions.

To date, only one randomized trial has been conducted to evaluate the impact of routine preoperative CAG on the results of CEA. In the previously mentioned study [20], this approach not only identified patients with asymptomatic obstructive coronary artery disease, but also allowed myocardial revascularization. This strategy led to a decrease in perioperative myocardial infarctions (OR 0.23; *p* = 0.01), as well as to an improvement in survival during further observation for 5 years (respectively, 95.6 ± 3.2% and 89.7 ± 3.7%; *p* = 0.01) [11]. In meta-analyses comparing the results of CEA and carotid artery stenting [25,26], the incidence of perioperative myocardial infarction in CEA is 1.5–2.2% (apparently, the incidence of concomitant coronary artery disease in such studies is low). On the other hand, in patients with combined lesions of the coronary and carotid arteries in some studies, the incidence of myocardial infarction during surgery reaches 11.5% [18]. It should be noted that myocardial revascularization before CEA made it possible to reduce the incidence of perioperative cardiac complications [12] down to a level comparable to that observed in patients without coronary artery disease in studies where such a comparison was carried out [27]. These data are quite consistent with our results. It should be noted that the detection of obstructive coronary artery disease before CEA allows the use of another treatment tactic—myocardial revascularization after CEA—which also has a favorable effect on the prognosis in such patients [22]. With a three-year follow-up, this made it possible to reduce the number of MACE (4% versus 17%), the number of cardiac deaths (2% versus 9%), the number of myocardial infarctions (3% versus 17%) and the number of deaths from cardiovascular diseases (2% versus 12%). In general, the determination of surgical tactics for combined lesions of the carotid and coronary arteries continues to be the subject of ongoing study [26,28,29]; the description of this issue is beyond the scope of this study.

The clinical significance of the study is that it confirms the need for routine CAG before CEA, supporting the opinion of Russian experts to upgrade the class of recommendations to IIA [6] instead of the IIB class proposed in the ESC guidelines [7]. The recently published ESC guidelines on chronic coronary syndrome do not address the issue of diagnosing coronary artery disease before CEA [15]. The problem is that the developed algorithm for diagnosing obstructive coronary artery lesions is aimed at identifying them in patients with symptoms of chest pain or dyspnea. The presence of peripheral artery disease (including carotid arteries) increases the clinical probability of coronary obstructive atherosclerosis [15]. However, asymptomatic coronary artery disease, which is often observed in carotid atherosclerosis, makes it impossible to use such a diagnostic algorithm. Other recent ESC guidelines (for the management of peripheral arterial and aortic diseases) [30] address the situation with the screening assessment of the state of the coronary arteries before CEA. They suggest the following: “… preoperative CAD screening, including coronary angiography, may be considered in suspected patients” [30]. Our data are quite consistent with this point of view, offering additional arguments for its validity. The data of the study by Kim et al. [31], in which the presence of severe coronary stenoses was associated with a higher frequency of perioperative complications (death, myocardial infarction, stroke), indicate that such diagnostic tactics may be appropriate in other countries. On the other hand, in patients with less severe cerebrovascular pathology with asymptomatic damage to the carotid arteries, other tactics of preoperative examination were encountered [32], although these surgeries are currently considered low-risk [7] which does not require any preoperative examination. The routine cardiologist consultation allowed the determination of indications for invasive CAG in 14.2%, while significant coronary stenoses were detected in 6.9% and myocardial revascularization was performed in 4.3% of cases. As a result, in the group with routine cardiologist consultation, it was possible to reduce the number of perioperative complications and improve the medium-term postoperative prognosis [32]. 

Another possible alternative may be to perform the CT angiography of the coronary arteries before surgery on the carotid arteries. Recently, CT angiography has been increasingly used for the non-invasive assessment of the coronary arteries [33]. In particular, the latest ESC guidelines for chronic coronary syndrome widely suggest using this diagnostic method in cases of low clinical probability of obstructive coronary artery disease [15]. Given that CT angiography is used to study the carotid arteries in patients, it is likely that coronary assessment could be performed during the same session. Despite the possible limitations of CT angiography of the coronary arteries (arrhythmias, high heart rates requiring beta-blocker therapy, renal impairment, severe coronary calcification), the first publications on its use before CEA confirm the feasibility of this approach. In the study by Uchida et al. [34], such an examination was performed in 81.1% of patients before CEA, and in 74.3% of patients before the stenting of the carotid arteries; coronary artery stenosis was detected in 32.6% and 41.8% of patients, respectively. In another study in patients with no symptoms of CAD undergoing CEA, preoperative coronary CT angiography and CT Fractional Flow Reserve analysis revealed silent coronary ischemia in 57% of cases [35].

When evaluating the results of this study, it is necessary to take into account the existing limitations. Firstly, this study is retrospective, conducted in one center, and therefore its results may be of limited applicability to other clinics. Secondly, in this study, all patients underwent CAG before CEA, which did not allow us to form a control group. Therefore, we cannot confidently judge the effectiveness of this strategy compared to CEA without preliminary CAG. Thirdly, we did not evaluate the dynamics of troponins in the perioperative period; therefore, we could not identify perioperative myocardial injury without signs of myocardial infarction. Finally, due to the small number of endpoints in the study, we were not able to strictly adhere to the number of “sufficient data” in the multivariate model [36]. This could potentially affect the results of the data analysis in this model, which is also a limitation of the study.

## 5. Conclusions

When conducting routine CAG during the examination of patients before CEA, obstructive coronary artery lesions were detected in 62% of cases, non-obstructive ones—in 23.7% intact coronary arteries—were detected in 14.3%. The groups differed significantly in the presence of symptoms of angina pectoris, myocardial infarction and myocardial revascularization procedures in the anamnesis and in the presence of stenosis of the arteries of the lower extremities. Myocardial revascularization before CEA or simultaneously with it was performed in 42.7% of patients. As a result, it was possible to reduce the number of perioperative cardiac complications (mortality 0.7%, perioperative myocardial infarction—1.9%), which is below the average figures for such operations. In multivariate analysis, independent predictors of MACE development were the presence of coronary artery disease, chronic kidney disease and the detection of obstructive coronary artery lesions. The high frequency of detection of obstructive coronary artery lesions in our patients and the minimal number of perioperative complications support the use of routine CAG before CEA.

## Figures and Tables

**Table 1:** Preoperative demographic data and clinical measurements.

Variable *n* (%)|Me (LQ;UQ)	Total (*n* = 498)	Group 1 with Obstructive CA (*n* = 309)	Group 2 with Non-Obstructive CA (*n* = 118)	Group 3 without Obstructive CA (*n* = 71)	*p* Value
Demographic Data					
Age	68.0 [63.0; 72.0]	68.0 [63.0; 72.0]	68.0 [64.0; 73.0]	66.0 [61.0; 70.0]	0.029 *p*_1–2_ = 0.352 *p*_1–3_ = 0.027 *p*_2–3_ < 0.001
Gender male	342 (68.7)	228 (73.8)	74 (62.7)	40 (56.3)	0.004 *p*_1–2_ = 0.022 *p*_1–3_ = 0.002 *p*_2–3_ = 0.371
Body mass index, kgm^–2^	27.5 [24.3; 30.4]	27.3 [24.2; 30.7]	27.5 [24.4; 30.1]	27.9 [24.8; 30.0]	0.988
City/village resident	67 (13.5)/ 431 (85.5)	43 (14.1)/ 266 (86.1)	17 (14.4)/ 101 (85.6)	7 (9.6)/ 64 (90.1)	0.626
Disability	59 (11.8)	42 (13.6)	11 (9.3)	6 (8.5)	0.301
Smoking history active	159 (31.9)	98 (31.7)	36 (30.5)	25 (35.2)	0.791
Smoking experience, years	40.0 [32.0; 50.0]	40.0 [32.0; 50.0]	40.0 [35.0; 50.0]	40.0 [20.0; 48.0]	0.439
Hypertension	431 (86.5)	273 (88.4)	100 (84.8)	58 (81.7)	0.268
Diabetes	87 (17.5)	59 (19.1)	16 (13.6)	12 (16.9)	0.399
Hyperlipidemia	437 (87.8)	270 (87.4)	107 (90.7)	60 (84.5)	0.432
Chronic cerebral ischemia	121 (24.3)	75 (24.3)	32 (27.1)	14 (19.7)	0.517
Stroke	199 (40.0)	117 (37.9)	52 (44.1)	30 (42.3)	0.461
Transischemic attack	15 (3.0)	10 (3.3)	3 (2.5)	2 (2.7)	0.918
Chronic kidney disease	50 (10.0)	29 (9.4)	13 (11.0)	8 (11.3)	0.822
COPD	13 (2.6)	8 (2.6)	3 (2.5)	2 (2.8)	0.992
Carotid endarterectomy	43 (8.6)	29 (9.4)	9 (7.6)	5 (7.0)	0.741
Oncological diseases	15 (3.0)	10 (3.2)	3 (2.5)	2 (2.8)	0.927
Cardiac History					
Coronary heart disease	295 (59.2)	219 (70.9)	61 (51.7)	15 (21.1)	<0.001 *p*_1–2_ < 0.001 *p*_1–3_ < 0.001 *p*_2–3_ < 0.001
Angina	190 (38.2)	136 (44.0)	44 (37.3)	10 (14.1)	<0.001 *p*_1–2_ = 0.211 *p*_1–3_ < 0.001 *p*_2–3_ < 0.001
Myocardial infarction	183 (36.7)	151 (49.0)	23 (19.5)	9 (12.7)	<0.001 *p*_1–2_ < 0.001 *p*_1–3_ < 0.001 *p*_2–3_ = 0.377
Previous PCI	94 (18.9)	79 (25.6)	15 (12.7)	0	<0.001 *p*_1–2_ < 0.001 *p*_1–3_ < 0.001 *p*_2–3_ < 0.001
Previous CABG	63 (12.7)	61 (19.8)	2 (1.7)	0	<0.001 *p*_1–2_ < 0.001 *p*_1–3_ < 0.001 *p*_2–3_ = 0.268
Peripheral vascular disease					
Carotid artery bilateral ≥ 30%	281 (56.4)	185 (60.3)	63 (53.4)	33 (45.2)	0.049 *p*_1–2_ = 0.198 *p*_1–3_ = 0.019 *p*_2–3_ = 0.274
Carotid artery bilateral ≥ 50%	156 (31.3)	106 (34.5)	35 (29.7)	15 (20.6)	0.062
Lower extremities ≥ 50%	81 (16.3)	62 (20.3)	13 (11.1)	6 (8.2)	0.009 *p*_1–2_ = 0.026 *p*_1–3_ = 0.015 *p*_2–3_ = 0.521
Lower-limb chronic ischaemia	64 (12.9)	47 (15.2)	12 (10.2)	5 (7.04)	0.109
Diagnostic studies					
Veloergometry	4 (0.8)	3 (1.0)	1 (0.9)	0	0.709
Stress echocardiography	5 (1.0)	4 (1.3)	1 (0.9)	0	0.603
Coronary CT angiography	40 (8.0)	27 (8.7)	10 (8.5)	3 (4.2)	0.442
Holter monitor	7 (1.4)	6 (1.9)	1 (0.9)	0	0.383
Prehospital medical treatment					
β-blockers	310 (62.2)	204 (66.0)	67 (56.8)	39 (54.9)	0.082
CCB	196 (39.4)	119 (38.5)	50 (42.4)	27 (38.0)	0.742
Statins	359 (72.1)	226 (73.1)	82 (69.5)	51 (71.8)	0.752
ARBs	159 (31.9)	95 (30.7)	42 (35.6)	22 (31.0)	0.619
ACEI	187 (37.6)	122 (39.5)	40 (33.9)	25 (35.2)	0.514
Aspirin	298 (59.8)	200 (64.7)	61 (51.7)	37 (52.1)	0.017 *p*_1–2_ = 0.015 *p*_1–3_ = 0.079 *p*_2–3_ = 0.818

Notes: CA—coronary arteries. COPD—chronic obstructive pulmonary disease. CABG—coronary artery bypass graft. PCI—percutaneous coronary intervention. ARBs—angiotensin II receptor blockers. ACEI—angiotensin-converting enzyme inhibitor. CCB—calcium channel blockers. *p*_1–2_—significant differences in pairwise comparison of groups 1 and 2. *p*_1–3_—significant differences in pairwise comparison of groups 1 and 3. *p*_2–3_—significant differences in pairwise comparison of groups 2 and 3.

**Table 2:** Characteristics of the routine laboratory parameters.

Variable Me (LQ;UQ)	Total (*n* = 498)	Group 1 with Obstructive CA (*n* = 309)	Group 2 with Non-Obstructive CA (*n* = 118)	Group 3 without Obstructive CA (*n* = 71)	*p* Value
Total cholesterol, mmol/L	3.9 [3.5; 4.9]	4.2 [3.6; 5.0]	3.7 [3.4; 4.1]	3.7 [3.5; 4.4]	0.336
HDL cholesterol, mmol/L	1.14 [0.9; 1.5]	1.07 [0.8; 1.47]	1.33 [1.29; 1.55]	1.0 [0.9; 1.13]	0.113
LDL cholesterol, mmol/L	2.1 [1.7; 3.0]	2.07 [1.65; 2.59]	2.0 [1.79; 2.27]	2.06 [2.01; 3.43]	0.669
Triglycerides, mmol/L	1.5 [1.1; 2.0]	1.5 [1.3; 2.2]	1.1 [0.7; 2.0]	1.35 [1.1; 1.7]	0.367
Creatinine, µmol/L	86.0 [73.0; 103.0]	85.0 [74.5; 100.5]	89.0 [73.0; 100.5]	85.5 [68.0; 113.5]	0.953
Glucose, mmol/L	6.4 [5.7; 7.3]	6.3 [5.7; 7.1]	6.8 [5.7; 7.8]	6.4 [5.7; 7.1]	0.356
Urea, Me (LQ;UQ) mmol/L	6.6 [5.3; 8.5]	6.6 [5.3; 8.3]	6.5 [5.35; 9.0]	7.4 [5.7; 10.7]	0.423

Notes: CA—coronary arteries. HDL—high-density lipoproteins. LDL—low-density lipoproteins.

**Table 3:** Preoperative echocardiography parameters.

Variable Me (LQ; UQ)	Total (*n* = 498)	Group 1 with Obstructive CA (*n* = 309)	Group 2 with Non-Obstructive CA (*n* = 118)	Group 3 without Obstructive CA (*n* = 71)	*p* Value
Aorta, cm	3.5 [3.3; 3.7]	3.6 [3.3; 3.7]	3.5 [3.3; 3.7]	3.5 [3.4; 3.6]	0.851
End-diastolic LV dimension, cm	5.2 [4.9; 5.5]	5.3 [5.0; 5.7]	5.1 [4.9; 5.3]	5.2 [5.0; 5.5]	<0.001 *p*_1–2_ < 0.001 *p*_1–3_ = 0.251 *p*_2–3_ = 0.048
End-systolic LV dimension, cm	3.4 [3.2; 3.8]	3.5 [3.2; 3.9]	3.2 [3.0; 3.5]	3.4 [3.1; 3.6]	<0.001 *p*_1–2_ < 0.001 *p*_1–3_ = 0.061 *p*_2–3_ = 0.066
End-diastolic LV volume, mL	124.0 [108.0; 142.0]	130.0 [108.0; 147.0]	118.0 [103.0; 135.0]	124.0 [108.0; 135.0]	0.028 *p*_1–2_ = 0.014 *p*_1–3_ = 0.118 *p*_2–3_ = 0.571
End-systolic LV volume, mL	47.0 [38.0; 58.0]	47.0 [38.0; 62.0]	41.0 [35.0; 51.0]	44.0 [35.0; 51.0]	0.023 *p*_1–2_ = 0.003 *p*_1–3_ = 0.017 *p*_2–3_ = 0.795
Left atrium, cm	4.3 [4.0; 4.6]	4.3 [4.0; 4.6]	4.2 [4.0; 4.6]	4.3 [4.0; 4.6]	0.415
LV ejection fraction %	63.0 [58.0; 65.0]	62.0 [55.0; 65.0]	65.0 [61.0; 66.0]	63.5 [60.0; 66.0]	<0.001 *p*_1–2_ < 0.001 *p*_1–3_ = 0.015 *p*_2–3_ = 0.193
IVST, cm	1.2 [1.0; 1.3]	1.2 [1.0; 1.3]	1.2 [1.0; 1.3]	1.1 [1.0; 1.2]	0.562
PW LV, cm	1.2 [1.0; 1.2]	1.2 [1.0; 1.3]	1.2 [1.0; 1.3]	1.1 [1.0; 1.2]	0.515
Right ventricular, cm	2.0 [1.8; 2.2]	2.0 [1.8; 2.2]	2.0 [1.85; 2.2]	2.0 [1.8; 2.4]	0.627
Right atrium, cm	4.0 [3.8; 4.6]	4.0 [3.8; 4.6]	4.0 [3.7; 4.4]	4.0 [3.8; 4.6]	0.161
mPAP, mmhg	27.0 [23.0; 30.0]	27.5 [23.0; 32.0]	26.0 [24.0; 29.0]	27.0 [23.0; 29.0]	0.580
E, cm/s	58.0 [48.0; 68.0]	58.0 [47.0; 68.0]	58.0 [50.0; 67.0]	54.5 [47.0; 70.5]	0.894
A, cm/s	73.5 [63.0; 84.0]	74.0 [63.0; 85.0]	75.5 [65.5; 87.0]	70.0 [61.5; 78.0]	0.261
E/A	0.74 [0.7; 0.9]	0.74 [0.66; 0.87]	0.73 [.067; 0.83]	0.73 [0.65; 1.25]	0.687

Notes: CA—coronary arteries. LV—left ventricular. EF—ejection fraction. IVST—interventricular septum thickness. PW LV—posterior wall of the left ventricle. mPAP—mean pulmonary arterial pressure. E—peak early diastolic left ventricular filling velocity. A—peak left ventricular filling velocity at atrial contraction. E/A—ratio of peak early diastolic filling velocity to peak filling velocity at atrial contract. *p*_1–2_—significant differences in pairwise comparison of groups 1 and 2. *p*_1–3_—significant differences in pairwise comparison of groups 1 and 3. *p*_2–3_—significant differences in pairwise comparison of groups 2 and 3.

**Table 4:** Perioperative outcomes.

Variable *n* (%)|Me (LQ;UQ)	Total (*n* = 498)	Group 1 with Obstructive CA (*n* = 309)	Group 2 with Non-Obstructive CA (*n* = 118)	Group 3 without Obstructive CA (*n* = 71)	*p* Value
Risk assessment of non-cardiac operations					
RCRI, %	2.0 [1.0; 2.0]	2.0 [2.0; 2.0]	2.0 [1.0; 2.0]	2.0 [1.0; 2.0]	<0.001 *p*_1–2_ < 0.001 *p*_1–3_ = 0.061 *p*_2–3_ = 0.066
NSQIP MICA, %	0.73 [0.63; 1.3]	0.72 [0.64; 1.3]	0.75 [0.61; 1.25]	0.69 [0.6; 1.3]	0.515
Simultaneous heart surgery and carotid endarterectomy					
Simultaneous surgery in total	45 (9.0)	42 (13.6)	2 (1.7)	1 (1.4)	<0.001
Carotid endarterectomy with CABG	15 (3.0)	15 (4.9)	0	0	0.008
Carotid endarterectomy with PCI	29 (5.8)	27 (8.7)	2 (1.7)	0	<0.001
Carotid endarterectomy with aortic valve replacement	1 (0.2)	0	0	1 (1.4)	0.05
Intraoperative characteristics					
Operation time, minutes	75.0 [65.0; 90.0]	75.0 [65.0; 95.0]	75.0 [65.0; 93.5]	72.5 [60.0; 90.0]	0.054
Intraoperative blood loss, mL	100.0 [50.0; 100.0]	100.0 [50.0; 100.0]	100.0 [50.0; 105.0]	100.0 50.0; 100.0]	0.604

Notes: CA—coronary arteries. NSQIP MICA—ACS National Surgical Quality Improvement Program Myocardial Infarction or Cardiac Arrest. RCRI—Revised Cardiac Risk Index. CABG—coronary artery bypass graft. PCI—percutaneous coronary intervention.

**Table 5:** Postoperative complications.

Variable *n* (%)	Total (*n* = 498)	Group 1 with Obstructive CA (*n* = 309)	Group 2 with Non-Obstructive CA (*n* = 118)	Group 3 without Obstructive CA (*n* = 71)	*p* Value
Mortality	2 (0.4)	2 (0.7)	0	0	0.541
Postoperative MI	6 (1.2)	6 (1.9)	0	0	0.156
Stroke	6 (1.2)	5 (1.6)	0	1 (1.4)	0.385
Arrhythmias: atrial fibrillation/flutter	8 (1.6)	8 (2.6)	0	0	0.083
MACCE	20 (4.0)	19 (6.2)	0	1 (1.4)	0.007 *p*_1–2_ = 0.005 *p*_1–3_ = 0.107 *p*_2–3_ = 0.201

Notes: CA—coronary arteries. MI—myocardial infarction. MACCE—major adverse cardiac and cerebrovascular event.

**Table 6:** Results of binary logistic regression (forward LR method): association of factors with the risk of combined endpoint development.

		B	S.E.	Wald	df	Sig.	Exp(B)	95% CI for Exp(B)	
								Lower	Upper
Step 1	Chronic kidney disease	1.490	0.562	7.035	1	0.008	4.436	1.475	13.339
	Constant	−3.407	0.293	134.802	1	0.000	0.033		
Step 2	Chronic kidney disease	1.623	0.576	7.927	1	0.005	5.066	1.637	15.676
	CAG Group	−1.456	0.689	4.469	1	0.035	0.233	0.060	0.899
	Constant	−1.574	0.818	3.700	1	0.054	0.207		
Step 3	Chronic kidney disease	1.850	0.604	9.393	1	0.002	6.358	1.948	20.750
	Coronary artery disease	−1.214	0.532	5.209	1	0.022	0.297	0.105	0.842
	CAG Group	−1.708	0.694	6.048	1	0.014	0.181	0.046	0.707
	Constant	−0.619	0.888	0.486	1	0.486	0.538		

## Data Availability

The datasets used and/or analyzed during the current study are available from the corresponding author on reasonable request.

## Supplementary Materials

The following supporting information can be downloaded at https://www.mdpi.com/article/10.3390/jcm13185495/s1, Table S1: Results of binary logistic regression (Enter method): association of factors with the risk of combined endpoint development, Table S2. Hosmer and Lemeshow test of binary logistic regression (Enter method): association of factors with the risk of combined endpoint development, Table S3. Omnibus Tests of Model Coefficients of binary logistic regression (forward LR method): association of factors with the risk of combined endpoint development, Table S4 Model Summary of binary logistic regression (forward LR method): association of factors with the risk of combined endpoint development, Table S5 Classification Table of binary logistic regression (forward LR method): association of factors with the risk of combined endpoint development, Table S6. Hosmer and Lemeshow test of multiple binary logistic regression (forward LR method): association of factors with the risk of combined endpoint development, Table S7 Receiver operating characteristic curve analysis. Performance of baseline parameters in discriminating combined endpoint development after carotid endarterectomy. Area under the curve.
