# Supplementary material for: A Routine Coronary Angiography before Carotid Endarterectomy as an Example of Interdisciplinary Work: The Immediate Results of the Surgery
Source: J Clin Med. 2024 Sep 17;13(18):5495. doi: 10.3390/jcm13185495 (PMC11432148; doi:10.3390/jcm13185495)
Supplement: Supplementary file 1 [file jcm-13-05495-s001.zip › jcm-3218913-supplementary.pdf]

Table S1. Results of binary logistic regression (Enter method): association of factors with the risk of combined endpoint development

							95% C.I.for EXP(B)	
	B	Lower	Upper	df	Sig.	Exp(B)	Lower	Upper
Age	0.079	0.985	1.189	1	0.101	1.082	0.985	1.189
Sex	-1.288	0.052	1.465	1	0.131	0.276	0.052	1.465
Chronic kidney disease	2.151	2.252	32.790	1	0.002	8.592	2.252	32.790
Coronary artery disease	-1.266	0.083	0.954	1	0.042	0.282	0.083	0.954
mPAP	-0.051	0.883	1.023	1	0.173	0.950	0.883	1.023
Right atrium	0.787	0.778	6.198	1	0.137	2.196	0.778	6,198
CAG Group	-1.855	0.034	0.729	1	0.018	0.156	0.034	0.729
ARB	-0.563	0.285	1.137	1	0.111	0.570	0.285	1.137
Statins	1.004	1.068	6.969	1	0.036	2.728	1.068	6.969
Constant	-7.677	3.906	3.863	1	0.049	0.000		

Notes: ARB - Angiotensin II receptor blocker, mPAP - mean pulmonary arterial pressure

Table S2. Hosmer and Lemeshow test of binary logistic regression (Enter method): association of factors with the risk of combined endpoint development

Step	Chi-square	df	Sig.
1	9.716	8	0.286

Table S3 Omnibus Tests of Model Coefficients of binary logistic regression (forward LR method): association of factors with the risk of combined endpoint development

		Chi-square	df	Sig.
Step 1	Step	5.732	1	0.017
	Block	5.732	1	0.017
	Model	5.732	1	0.017
Step 2	Step	7.872	1	0.005
	Block	13.604	2	0.001
	Model	13.604	2	0.001
Step 3	Step	5.210	1	0.022
	Block	18.814	3	0.000
	Model	18.814	3	0.000

Table S4 Model Summary of binary logistic regression (forward LR method): association of factors with the risk of combined endpoint development

Step	-2 Log likelihood	Cox & Snell R Square	Nagelkerke R Square
1	136.026	0.014	0.047
2	128.154	0.032	0.112
3	122.944	0.045	0.153

Table S5 Classification Table of binary logistic regression (forward LR method): association of factors with the risk of combined endpoint development

	Observed		Predicted		
			Combined endpoint development		
			0	1	Percentage Correct
Step 1	Combined endpoint development	0	396	0	100.0
		1	17	0	0.0
		Overall Percentage			95.9
Step 2	Combined endpoint development	0	396	0	100.0
		1	17	0	0.0
		Overall Percentage			95.9
Step 3	Combined endpoint development	0	396	0	100.0
		1	17	0	0.0
		Overall Percentage			95.9

Table S6. Hosmer and Lemeshow test of multiple binary logistic regression (forward LR method): association of factors with the risk of combined endpoint development

Step	Chi-square	df	Sig.
1	,000	0	.
2	7,019	3	,071
3	8,177	6	,225

Table S7 Receiver operating characteristic curve analysis. Performance of baseline parameters in discriminating combined endpoint development after carotid endarterectomy. Area under the curve.

Test Result Variable(s)				Asymptotic 95% Confidence Interval	
	Area	Std. Error	Asymptotic Sig	Lower Bound	Upper Bound
Chronic kidney disease	0.604	0.072	0.115	0.463	0.745
Coronary artery disease	0.426	0.066	0.261	0.297	0.555
CAG Group	0.665	0.051	0.012	0.565	0.766
